# Use of the Internet to Communicate with Health Care Providers in the United States: Estimates from the 2003 and 2005 Health Information National Trends Surveys (HINTS)

**DOI:** 10.2196/jmir.9.3.e20

**Published:** 2007-07-12

**Authors:** Ellen Burke Beckjord, Lila J Finney Rutten, Linda Squiers, Neeraj K Arora, Lindsey Volckmann, Richard P Moser, Bradford W Hesse

**Affiliations:** ^5^Division of Cancer Control and Population Sciences (DCCPS)Behavioral Research Program (BRP)National Cancer Institute (NCI)National Institutes of Health (NIH)BethesdaMDUSA; ^4^Division of Cancer Control and Population Sciences (DCCPS)Applied Research ProgramOutcomes Research BranchNational Cancer Institute (NCI)National Institutes of Health (NIH)BethesdaMDUSA; ^3^Cancer Information ServiceNational Cancer Institute (NCI)National Institutes of Health (NIH)BethesdaMDUSA; ^2^Division of Cancer Control and Population Sciences (DCCPS)Behavioral Research Program (BRP)Health Communication and Informatics Research Branch (HCIRB)National Cancer Institute (NCI)National Institutes of Health (NIH)BethesdaMDUSA; ^1^Cancer Prevention Fellowship ProgramDivision of Cancer PreventionOffice of Preventive OncologyNational Cancer Institute (NCI)National Institutes of Health (NIH)BethesdaMDUSA

**Keywords:** Internet, patient-provider communication, electronic mail, information services, trends and utilization, medical informatics, trends, health education, health services, demography, data collection, health care surveys, neoplasms, regression analysis

## Abstract

**Background:**

Despite substantial evidence that the public wants access to Internet-based communication with health care providers, online patient-provider communication remains relatively uncommon, and few studies have examined sociodemographic and health-related factors associated with the use of online communication with health care providers at a population level.

**Objective:**

The aim of the study was to use nationally representative data to report on the prevalence of and changes in use of online patient-provider communication in 2003 and 2005 and to describe sociodemographic and health-related factors associated with its use.

**Methods:**

Data for this study are from two iterations of the Health Information National Trends Survey (HINTS 2003, HINTS 2005). In both years, respondents were asked whether they had ever used email or the Internet to communicate with a doctor or a doctor’s office. Adult Internet users in 2003 (n = 3982) and 2005 (n = 3244) were included in the present study. Multivariate logistic regression analysis was conducted to identify predictors for electronic communication with health care providers.

**Results:**

In 2003, 7% of Internet users had communicated online with an health care provider; this prevalence significantly increased to 10% in 2005. In multivariate analyses, Internet users with more years of education, who lived in a metro area, who reported poorer health status or who had a personal history of cancer were more likely to have used online patient-provider communication.

**Conclusions:**

Despite wide diffusion of the Internet, online patient-provider communication remains uncommon but is slowly increasing. Policy-level changes are needed to maximize the availability and effectiveness of online patient-provider communication for health care consumers and health care providers. Internet access remains a significant barrier to online patient-provider communication.

## Introduction

For more than a decade, studies have consistently shown that some members of the public want access to Internet-based communication with health care providers, with preference estimates for online patient-provider communication ranging from 40% to 83% [1-9]. The Institute of Medicine has characterized patient-centered care as health care that addresses patient needs and preferences, and it has identified information technology as crucial to advancing health care quality [10]. There is evidence that online communication with health care providers promotes health-related quality of life (eg, [11]) and, further, that health care consumers would benefit from increased partnerships between health information technology and health care providers: health care providers are a more trusted source of health information than the Internet, but the Internet is the source most often used by the public to retrieve health information [12]. Thus, as the presence of eHealth applications such as online patient-provider communication grows within the health care landscape, it is important to examine the prevalence of Internet-based patient-provider communication and to identify sociodemographic and health-related factors associated with its use to ensure that the potential benefits of online communication with health care providers are available to all health care consumers.

Despite the penetration of Internet access (an estimated 73% of American adults are online, 91% of whom use email [13]) and the growing public endorsement of the Internet as a useful tool in health-related decision making [14], online patient-provider communication remains uncommon [15-18]. While acknowledging potential benefits of online patient-provider communication (eg, for scheduling appointments [19]), health care providers have concerns regarding confidentiality, reimbursement, and workload related to online communication with patients [20]. In general, preference for online communication is higher among patients than among health care providers [21].

In 2003, the National Cancer Institute launched the first biennial Health Information National Trends Survey (HINTS) [22,23]. HINTS was designed to capture nationally representative data on the impact of the cancer information environment and to specifically assess the public’s use of information technology for health. Though previous estimates of the online public’s use of the Internet to communicate with health care providers have been reported from nationally representative data [15,17,24], the purpose and design of HINTS allow for a more in-depth examination of sociodemographic and health-related factors in relation to online communication with health care providers than has been possible in previous investigations. Further, using cross-sectional iterations of HINTS from 2003 and 2005, we can examine whether the prevalence of online communication with health care providers among American Internet users has changed over time.

In 2001, an estimated 6% of Internet users had emailed health care providers [15], up to 7% in 2003 [17]. In this paper, we provide estimates of Internet-based patient-provider communication derived from national probability samples of online American adults in 2003 and 2005. In addition, we report on changes in use of the Internet to communicate with health care providers over this 2-year period and identify sociodemographic and health-related factors associated with online patient-provider communication. Overall, we expect that the prevalence of online patient-provider communication will be low, but that use of the Internet to communicate with health care providers will significantly increase between 2003 and 2005. Further, based on results of previous studies [2,25], we expect that Internet users with more years of education, who are of higher socioeconomic status, and who are more engaged with the health care system will be more likely to report having communicated online with an health care provider.

## Methods

### Data Source

Data for this study are from HINTS 2003 and HINTS 2005, two iterations of the nationally representative survey designed to assess the impact of the cancer information environment and the public’s knowledge of, attitudes toward, and behaviors related to cancer and cancer prevention [26]. Comprehensive reports on the conceptual framework of HINTS and sample designs are published elsewhere [22,23].

 Data for HINTS 2003 were collected from October 2002 through April 2003. Data for HINTS 2005 were collected from February through August 2005. The cross-sectional surveys were administered by trained interviewers to representative samples of American households drawn from all telephone exchanges in the United States. Exchanges with high numbers of African Americans and Hispanics were oversampled in 2003. One adult (age 18 or older) was selected from each household to participate in the full survey during a household screening. In 2003, response rates were 55% at the household screening level (ie, the initial contact with the household used for sampling purposes) and 63% at the sampled person interview level (ie, completion of the interview by the sampled household member); in 2005, the respective response rates were 34% and 61%. Every sampled adult who completed a questionnaire in HINTS 2003 and HINTS 2005 was assigned a final sampling weight and a set of 50 replicate sampling weights. These sampling weights were used for the purpose of computing nationally representative estimates, to adjust for nonresponse and to reduce the sampling variance of estimators through utilization of information with less sampling and nonsampling error than the corresponding HINTS estimates (eg, estimates obtained through the Current Population Survey, which has much larger sample sizes than HINTS).

Complete interviews were conducted with 6369 adults for HINTS 2003 and with 5394 adults for HINTS 2005. In both surveys, only Internet users (n = 3982 in 2003; n = 3244 in 2005) were asked whether they had used email or the Internet to communicate with a doctor or a doctor’s office in the past 12 months (yes/no). Thus, Internet users in HINTS 2003 and 2005 served as the study populations for the current investigation.

### Study Variables

As in prior investigations of Internet users’ use of online patient-provider communication (eg, [15]), sociodemographic variables included in the present study were age, gender, education, annual income, race/ethnicity, and metropolitan statistical area (metro or non-metro county). Previous studies have also suggested that health-related variables, such as poorer self-reported health status [2] and having health insurance [25], are associated with use of online patient-provider communication. Health-related variables included in the present study were self-reported health status, possession of health insurance, and personal cancer history.

### Data Analysis

Analyses were conducted using SAS-callable SUDAAN, version 9.0 [27] to account for the complex survey design of HINTS and to obtain appropriate standard errors and 95% confidence intervals (CIs) for point estimates. Responses of “refused” or “don’t know” were counted as missing. Unweighted and weighted descriptive statistics are presented, and weighted data were used in all inferential statistical analyses. Bivariate analyses (chi-square) estimated changes in the prevalence of online communication with health care providers between 2003 and 2005 and associations between sociodemographic or health-related factors with online communication in 2003 and 2005. Three multivariate logistic regression models were used to estimate the odds of having used online communication with health care providers. The first used the combined data set of Internet users from 2003 and 2005 to model changes in use of online patient-provider communication over this 2-year period. We then examined sociodemographic and health-related factors separately in 2003 and 2005 to determine whether study variables associated with use of online patient-provider communication were consistent over time. The regression models used a forced-entry variable selection wherein all study variables were entered in one step.

## Results

### Sample Characteristics and Bivariate Analyses

Table 1 displays the sample characteristics of Internet users in HINTS 2003 and HINTS 2005 (weighted and unweighted). In 2003, 7% of Internet users reported communicating online with an health care provider in the past 12 months, consistent with previous prevalence estimates of Internet-based patient-provider communication in 2003 [17]. In 2005, 10% of Internet users reported communicating online with an health care provider. In bivariate analyses, this increase in use of Internet-based patient-provider communication from 2003 to 2005 was statistically significant (*χ* ^2^_1_ = 9.44; *P* = .003).

Bivariate associations between study variables and emailing health care providers are displayed in Table 2. In 2003, respondents who had communicated online with an health care provider had significantly more years of education and were more likely to reside in a metro county. In 2005, they were more likely to be female, had significantly more years of education, and were more likely to have a personal history of cancer.

**Table 1:** Sample characteristics of Internet users in HINTS 2003 and HINTS 2005^*^

	% of HINTS 2003 Internet Users (n = 3982)		% of HINTS 2005 Internet Users (n = 3244)	
	Unweighted	Weighted	Unweighted	Weighted
Communicated online with an health care provider in the past 12 months	8	7	10	10
**Age (years)**				
18-34	32	38	24	38
35-49	37	36	33	33
50-64	23	20	30	21
65-74	6	4	9	5
75 or older	2	2	4	2
**Gender**				
Male	41	50	36	48
Female	59	50	64	52
**Education**				
Less than high school	4	7	4	5
High school graduate	22	25	19	24
Some college	31	33	32	38
College graduate	44	35	45	33
**Annual Income (US $)**				
< 25000	17	16	12	13
25000 to < 35000	12	11	8	7
35000 to < 50000	18	18	13	12
50000 to < 75000	22	22	22	21
75000 or more	31	33	31	33
**Race/Ethnicity**				
White	73	75	82	77
Hispanic/Latino	8	7	6	8
African American	10	8	7	9
Asian American/Other^†^	5	6	6	7
**Health Insurance**				
No	9	11	9	12
Yes	90	89	91	88
**Health Status**				
Excellent/very good/good	84	84	85	84
Fair/poor	15	16	15	16
**History of Cancer**				
Yes	10	8	12	9
No	90	92	88	91
**Metropolitan Statistical Area**				
County in metro area	84	84	81	82
County in non-metro area	16	16	19	18

^*^Within-category cell values that add up to less than 100% reflect missing data due to responses of “refused” or “don’t know.”

^†^Other includes Pacific Islander, Native Hawaiian, American Indian, Alaska Native, and multiple races mentioned.

**Table 2:** Bivariate associations between communicating online with health care providers and study variables in HINTS 2003 and HINTS 2005

	HINTS 2003		HINTS 2005	
	% Communicated online with an health care provider ^*^	*P*^†^	% Communicated online with an health care provider^*^	*P*^†^
**Age (years)**		.19		.35
18-34	6.4		10.2	
35-49	6.8		9.3	
50-64	9.3		10.1	
64-75	4.6		6.4	
75 or older	6.7		7.0	
**Gender**		.19		.02
Male	7.6		7.9	
Female	6.4		11.2	
**Education**		< .001		.049
Less than high school	3.2		8.3	
High school graduate	3.5		6.6	
Some college	7.3		10.1	
College graduate	10.3		11.7	
**Annual Income (US $)**		.07		.17
< 25000	7.9		7.6	
25000 to < 35000	6.5		9.9	
35000 to < 50000	5.5		7.6	
50000 to < 75000	6.5		8.4	
75000 or more	9.2		12.8	
**Race/Ethnicity**		.88		.34
White	7.3		9.5	
Hispanic/Latino	6.4		5.9	
African American	6.1		11.3	
Asian American/Other^‡^	7.2		13.5	
**Health Insurance**		.54		.27
No	7.3		10.1	
Yes	6.3		7.8	
**Health Status**		.21		.79
Excellent/very good/good	6.9		9.9	
Fair/poor	8.3		9.3	
**History of Cancer**		.13		.03
Yes	9.4		14.5	
No	6.8		9.1	
**Metropolitan Statistical Area**		< .001		.12
County in metro area	7.5		10.1	
County in non-metro area	4.3		7.4	

^*^Weighted percents of Internet users who communicated online with an health care provider in the past 12 months within each study variable category.

^†^From chi-square tests (with degrees of freedom equaling number of categories minus 1).

^‡^Other includes Pacific Islander, Native Hawaiian, American Indian, Alaska Native, and multiple races mentioned.

### Multivariate Analyses

Our first multivariate logistic regression estimated changes in the odds of having communicated online with an health care provider between 2003 and 2005. This analysis was done with a combined data set of Internet users from HINTS 2003 and 2005 (n = 7134). The year of HINTS administration (2003 or 2005) was included to examine whether the increase in prevalence of online patient-provider communication remained significant after adjustment for the study variables in Table 1 (data not shown). Consistent with bivariate results, the increase in prevalence of online patient-provider communication among adult Internet users between 2003 and 2005 was significant; there was a 33% increase in the odds of having communicated online with an health care provider among respondents in HINTS 2005 compared to respondents in HINTS 2003 (OR = 1.33; 95% CI = 1.04-1.70; *P* = .03).

Table 3 displays the results of the multivariate analyses by HINTS year. Consistent with bivariate results, in 2003, education and metropolitan statistical area were associated with use of online patient-provider communication. Specifically, Internet users who were college graduates had over three times the odds of communicating online with an health care provider compared to those with less than a high school education (OR = 3.73; 95% CI = 1.10-12.59; *P* = .03). Those who lived a non-metro area were less likely to have used online patient-provider communication compared to Internet users who resided in metro area counties (OR = 0.62; 95% CI = 0.41-0.95; *P* = .03). Finally, Internet users who reported “fair” or “poor” health status had higher odds of communicating online with an health care provider (OR = 1.46; 95% CI = 1.00-2.04; *P* = .05).

For Internet users in 2005, women were more likely to have communicated online with an health care provider compared to men (OR = 1.47; 95% CI = 1.00-2.15; *P* = .05), and cancer survivors were more likely to have used online patient-provider communication compared to those without a history of cancer (OR = 1.99; 95% CI = 1.27-3.12; *P* = .002). These results are consistent with bivariate analyses; however, education was not associated with online patient-provider communication in the multivariate model.

**Table 3:** Multivariate logistic regressions of having used online patient-provider communication in HINTS 2003 or HINTS 2005

	Odds of Communicating Online With an health care provider in the Past 12 Months			
	HINTS 2003 (n = 3527)		HINTS 2005 (n = 2649)	
	OR (95% CI)	P^*^	OR (95% CI)	P^*^
**Age (years)**		.33		.35
18-34	1.00		1.00	
35-49	0.82 (0.55-1.23)	.33	0.77 (0.44-1.33)	.34
50-64	1.14 (0.76-1.70)	.53	0.76 (0.41-1.42)	.38
65-74	0.57 (0.26-1.23)	.15	0.45 (0.18-1.13)	.09
75 or older	1.07 (0.30-3.77)	.91	0.47 (0.15-1.51)	.20
**Gender**				
Male	1.00		1.00	
Female	0.75 (0.56-1.02)	.07	1.47 (1.00-2.15)	.05
**Education**		< .001		.26
Less than high school	1.00		1.00	
High school graduate	1.20 (0.34-4.31)	.77	0.56 (0.16-1.95)	.35
Some college	2.44 (0.71-8.42)	.16	0.93 (0.26-3.35)	.91
College graduate	3.73 (1.10-12.59)	.03	0.99 (0.28-3.48)	.99
**Annual Income (US $)**		.45		.34
< 25000	1.00		1.00	
25000 to < 35000	0.75 (0.36-1.55)	.42	1.35 (0.53-3.44)	.52
35000 to < 50000	0.59 (0.31-1.14)	.11	0.95 (0.49-1.88)	.89
50000 to < 75000	0.65 (0.37-1.16)	.14	1.09 (0.62-1.90)	.76
75000 or more	0.78 (0.44-1.36)	.37	1.56 (0.86-2.81)	.14
**Race/Ethnicity**		.98		.62
White	1.00		1.00	
Hispanic/Latino	0.92 (0.49-1.75)	.80	0.53 (0.16-1.72)	.28
African American	0.92 (0.53-1.59)	.75	1.26 (0.63-2.55)	.50
Asian American/Other^†^	1.00 (0.46-2.16)	.99	1.05 (0.43-2.58)	.91
**Health Insurance**				
Yes	1.00		1.00	
No	1.00 (0.52-1.90)	.99	0.99 (0.51-1.90)	.96
**Health Status**				
Excellent/very good/good	1.00		1.00	
Fair/poor	1.43 (1.00-2.04)	.05	0.88 (0.56-1.39)	.58
**History of Cancer**				
No	1.00		1.00	
Yes	1.34 (0.85-2.13)	.21	1.99 (1.27-3.12)	.002
**Metropolitan Statistical Area**				
County in metro area	1.00		1.00	
County in non-metro area	0.62 (0.41-0.95)	.03	0.76 (0.49-1.18)	.21

^*^ *P* values reported for category headings for study variables with more than 2 categories refer to main effects.

^†^Other includes Pacific Islander, Native Hawaiian, American Indian, Alaska Native, and multiple races mentioned.

## Discussion

Despite over a decade of research and the availability of guidelines for use of Internet-based communication by health care providers [28], the number of health care consumers using online patient-provider communication is still far below estimates of the number who would prefer to do so. Though data from HINTS suggest that use is slowing increasing, diffusion of online patient-provider communication is occurring at a pace far slower than diffusion of Internet use in general [29].

Thus, the question remains: Why is the overall prevalence of online communication with health care providers so low? While health care consumers and health care providers express concerns about communicating online, ratings of satisfaction and predictions about impact on health care quality regarding Internet-based communication have been generally favorable among both health care consumers and health care providers (eg, [5,30,31]). Therefore, use of online patient-provider communication will likely not significantly increase through efforts to change the primarily positive attitudes of health care consumers or health care providers, but rather, through changes in policies related to health care delivery [32] and through development of systems that prioritize usability [33]. Recent increased availability and adoption of online personal health records and electronic health records will likely affect the prevalence of online patient-provider communication [34,35], as will policies at the state and federal levels designed to promote diffusion of health information technology (eg, [36]). Continued implementation of policies that provide an architecture of support for online patient-provider communication and that address issues related to consumer and health care provider preferences, system interoperability, data security, and health care costs will be critical for maximizing the availability, adoption, and effectiveness of Internet-based communication between health care consumers and health care providers [32,33,35,37,38].

Associations between Internet users’ sociodemographic and health-related characteristics and use of online patient-provider communication reveal insights regarding who may be taking the lead with online health care provider communication and who may be left behind. In 2003, Internet users with high levels of education were more likely to have communicated online with an health care provider, consistent with previous studies [5]. That education was nonsignificant in 2005 may suggest that health care consumers’ level of education is less of a barrier to communicating online with health care providers as the prevalence of online patient-provider communication increases. Similarly, though Internet users residing in non-metro counties were less likely to have used online patient-provider communication in 2003, metropolitan statistical area was not associated with use in 2005. Deeper penetrance of high-speed Internet access into more rural areas [39,40] may have decreased, over time, the degree to which location prevented online communication with health care providers. In both years, indicators of poorer health status (poor/fair self-reported health status, personal cancer history) were associated with online health care provider communication, suggesting that Internet users with more medical problems or who are more engaged with the health care system due to a significant medical history may be more “hooked in” to Internet-based health communication resources or may have more a frequent need to use them. Finally, in 2005, women were more likely to use online patient provider communication compared to men. This result is consistent with findings that online women are more likely to search specifically for health information compared to men [41] and that higher percentages of women use the Internet for interpersonal communication related to health (eg, use of online support groups or health-based chat rooms [42]).

We did not observe associations between online communication with health care providers and characteristics such as race/ethnicity or annual income that have been documented in other studies (eg, [2,25]) as evidence of a “digital divide” [43,44]. Nonetheless, research and policy should continue to address groups potentially affected by the digital divide to ensure that advances in health information technology benefit all health care consumers [45]. Finally, our results were not consistent with previous studies that observed younger Internet users to be more likely to engage in online communication with health care providers (eg, [2,6]), suggesting a potential growth in comfort with online communication among Internet users of all ages.

### Limitations

Though HINTS data are nationally representative, the generalizability of our results may be limited by survey response rates and the drop in response rates between 2003 and 2005. However, HINTS response rates are comparable to those of other national random digit dial surveys [46], and the agreement between our findings regarding prevalence of online patient-provider communication with other reports [17] supports the reliability of the HINTS estimates. Further, estimates of Internet penetration vary widely in the published literature; HINTS penetrance estimates may be more conservative than data reported through market analysis firms due to the degree of sampling precision mandated for federal surveys that provide publicly available data. Due to item wording, we can only discuss our results at a generalized level of “online patient-provider communication” or “Internet-based communication with health care providers” and cannot characterize this behavior in more specific ways (eg, use of personal email, use of a Web portal) that could potentially affect our findings and resulting conclusions [38]. Finally, though HINTS provides a valuable population-level perspective on the prevalence of Internet-based health care provider communication and information on the characteristics of those who use it, all data are based on self-report, and HINTS does not allow for more in-depth examinations of barriers to communicating online with health care providers or the perceived benefits for those who do. To best meet the needs of patients and health care providers, research should continue to assess health care consumers’ and health care providers’ perspectives on barriers and benefits related to use of Internet-based communication as health information technology increasingly becomes part of standard medical care.

### Conclusions

Online patient-provider communication is increasing slowly but remains uncommon. Though lower levels of education and non-metro county residence may have been barriers to using Internet-based communication with health care providers in 2003, by 2005, these barriers were not evident in HINTS. However, use of online patient-provider communication is higher among Internet users who are experiencing health problems or who have significant medical histories; health care consumers without specific medical issues may need increased prompting to use Internet-based communication with health care providers as they engage in preventive health care. Changes in health care policy will be necessary to increase diffusion and adoption of online patient-provider communication, and a significant barrier continues to be Internet access. Disparities in Internet access must be addressed to ensure that increasing use of online patient-provider communication does not widen the digital divide or amplify disparities in health care quality for the underserved and underrepresented [25,45].

## Abbreviations

HINTS: Health Information National Trends Survey
